# Case Report: Staged epicardial pacemaker implantation in a 740 g ELBW infant with 25 + 2 weeks of GA with congenital complete heart block

**DOI:** 10.3389/fped.2025.1626650

**Published:** 2025-07-16

**Authors:** Tamari Tvildiani, Teresa Riedl-Seifert, Markus Waitz, Thomas Dimpfl, Thomas Paul, Andreas Jenke

**Affiliations:** ^1^ Department of Cardiac Surgery, Hospital Kassel, Kassel, Germany; ^2^ Department of Neonatology and Paediatric Gastroenterology, Children's Hospital Kassel, Kassel, Germany; ^3^ Department of Obstetrics and Gynaecology, Hospital Kassel, Kassel, Germany; ^4^ Department of Pediatrics, Faculty of Health, University of Witten/Herdecke, Witten, Germany

**Keywords:** heart block, pacemaker, premature infant, autoimmune etiology, extremely low birth weight (ELBW) infant

## Abstract

**Background:**

Congenital complete atrioventricular (AV) block is a rare but potentially fatal condition in neonates, especially those with extremely low birth weight (ELBW). Management in this population is challenging due to technical limitations and high comorbidity risk.

**Case presentation:**

We report the case of a female infant born at 25 + 2 weeks' gestation, weighing 740 g, with immune-mediated congenital complete AV block. Temporary epicardial pacing was initiated on day one due to persistent bradycardia and hemodynamic compromise. A dual-electrode strategy with alternating pacing sites maintained low thresholds for 90 days. A permanent pacemaker was implanted at 2,890 g. Despite complications including hydrops fetalis, intraventricular hemorrhage, hydrocephalus, and intestinal perforation, the patient was discharged in good condition.

**Conclusion:**

This case demonstrates the feasibility of staged pacemaker implantation in ELBW infants and supports individualized pacing strategies to optimize outcomes in this vulnerable population.

## Introduction

Congenital complete atrioventricular (AV) block is an uncommon but potentially life-threatening neonatal conduction disorder. It may occur in association with structural cardiac defects or, more frequently in isolated cases, as a consequence of maternal transplacental passage of anti-SSA/Ro or anti-SSB/La antibodies. The estimated incidence is approximately 1 in 20,000 live births, with fetal mortality rates ranging between 11% and 25% (1–3).

In neonates with complete AV block, permanent pacing is often required. Advances in surgical technique and pacemaker miniaturization have facilitated successful implantation in very small infants. However, in extremely low birth weight (ELBW) preterm neonates, particularly those born before 28 weeks of gestation, pacing remains a formidable clinical challenge due to the risks of infection, poor tissue resilience, high comorbidity burden, and technical limitations in lead and generator placement (4, 5).

To date and to our best knowledge, according to the literature only five cases of permanent pacemaker implantation in infants born between 26 and 28 weeks of gestation have been reported (6–9). Among these, two patients did not survive the neonatal period. The smallest previously reported surviving patient was born at 30 weeks' gestation and weighed 808 g (10), underscoring the rarity and difficulty of such interventions at earlier gestational ages (Table 1).

**Table 1:** Previously reported cases of permanent pacemaker implantation in infants born before 29 weeks’ gestation.

Year	Gestational week (weeks)	Birth weight (g)	Heart rate at birth (/min)	Age at operation for initial pacing (days)	Initial pacemaker type	Outcome
2000 (6)	26	830	50	0	Transthoracic	Dead
2007 (7)	27	980	55	2	Epicardial	Alive
2012 (8)	28	650	Not documented	1	Permanent	Dead
	28	840	Not documented	1	Permanent	Alive
2024 (9)	27	850	48	0	Epicardial	Alive
Our case	25	740	46	1	Epicardial	Alive

In this report, we describe the case of an infant born at 25 + 2 weeks' gestation with a birth weight of 740 g—currently the most premature and lowest-weight patient known to have undergone successful staged epicardial pacemaker implantation. Our case illustrates the feasibility of temporary pacing as a bridging strategy to definitive implantation in an ELBW neonate and highlights the potential for individualized approaches in neonatal pacing care.

## Case presentation

A 33-year-old woman (gravida 3, para 0) was admitted to our hospital as an emergency referral at 25 + 2 weeks' gestation due to pathological cardiotocography revealing persistent fetal bradycardia. Her obstetric history was notable for two prior late miscarriages at 20 and 21 weeks of gestation. The current pregnancy had been uneventful until the time of referral. On admission, the fetus demonstrated significant bradycardia and clear clinical signs of hydrops fetalis, including generalized edema and ascites, indicative of impending cardiac decompensation. Given the critical fetal condition, the past medical history of the woman and lack of sufficient time for antenatal corticosteroid administration, an immediate emergency cesarean section was performed. A female infant was delivered, weighing 740 g, with Apgar scores of 6, 8, and 8 at 1, 5, and 10 min, respectively. Umbilical cord arterial pH was 7.22. Postnatally, the infant exhibited signs of mild hydrops fetalis, including low-grade generalized edema and ascites. Due to worsening respiratory distress, initial CPAP support (FiO₂ up to 60%) was escalated to endotracheal intubation and administration of exogenous surfactant (Curosurf).

Continuous monitoring revealed a heart rate and peripheral pulse at approximately 70/min, subsequently decreasing to 50/min. Despite administration of Epinephrine, Calcium-gluconate, and Hydrocortisone, bradycardia persisted. The diagnosis of congenital complete AV block was established, and the patient was transferred to the neonatal intensive care unit.

**Figure 1:**
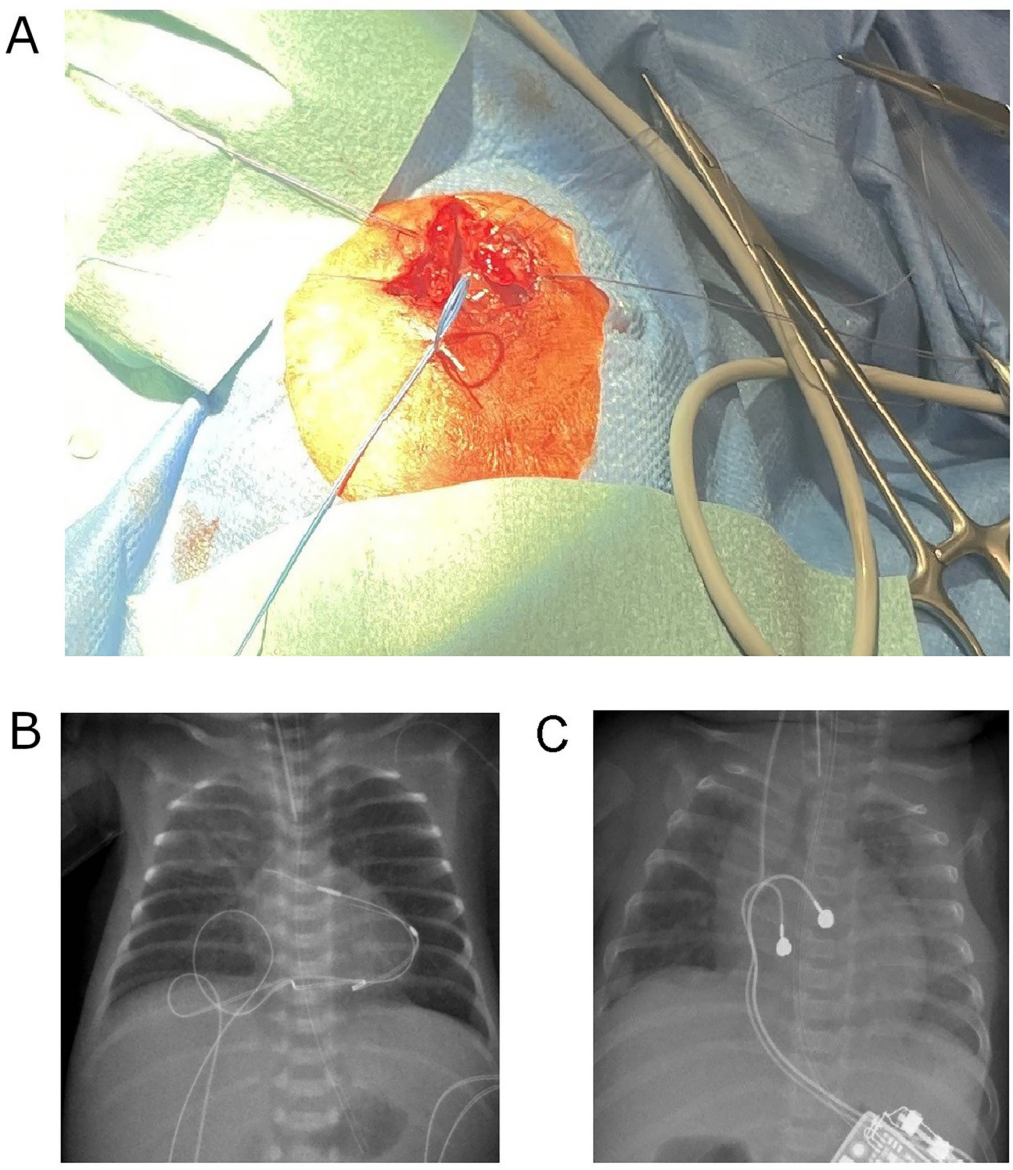
**(A)** Intraoperative image showing the placement of two epicardial pacing electrode pairs on the right ventricular surface (ventral and caudal sites) in the ELBW infant. **(B)** Postoperative chest and abdominal x-ray on day 5 of life showing the dual-site temporary pacing strategy, with two sets of epicardial pacing wires positioned at distinct ventral and caudal locations. **(C)** Postoperative chest and abdominal x-ray on day 90 of life demonstrating the permanent pacemaker generator positioned in the left upper abdominal quadrant and the bipolar lead attached to the right ventricular diaphragm and free wall.

At 16 h of life, due to ongoing hemodynamic instability. persisting bradycardia at 45/min, lactatemia and poor peripheral perfusion despite pharmacological therapy, temporary epicardial pacing wires were implanted at the right ventricular myocardium via a partial inferior sternotomy by the cardiothoracic surgery team. Importantly, we decided to implant two sets of epicardial pacing wires at different locations (ventral and caudal, Figures 1A,B). This allowed for alternate usage of the electrode pairs every 3–5 days, thereby preventing local fibrosis and maintaining myocardial capture. External ventricular pacing was initiated at a rate of 130–140 min at 2.8 mV, 0.5 msec. Postoperative stabilization was supported by Epinephrine infusion with 0.2 µg/kg/min. A hemodynamically significant patent ductus arteriosus (PDA) was closed by ibuprofen administration.

On day 3, cranial ultrasound showed an intraventricular hemorrhage (IVH) grade III on the right without parenchymal involvement and grade I on the left side, respectively. Subsequently, posthemorrhagic hydrocephalus developed necessitating ventricular reservoir insertion on day 37. Serial cerebrospinal fluid (CSF) withdrawals were performed subsequently without complications. On day 112, a permanent ventriculoperitoneal (VP) shunt was successfully implanted, also without complications.

On day 8, she developed acute abdominal distension with radiologic evidence of free intraabdominal air. Despite initial conservative management using peritoneal drainage and antibiotics, open laparotomy was inevitable on day 13, revealing an isolated focal intestinal perforation 12 cm proximal to the Bauhin's valve. A short intestinal segment was resected, and a double-barreled ileostomy was created. Uncomplicated reanastomosis of the gut was performed on day 47 of life. Parenteral nutrition was weaned off, and she reached full enteral autonomy on day 61 of life.

Immunological testing of the mother revealed elevated levels of anti-SSA/Ro antibodies, anti-52 kDa SSA/Ro antibodies and anti-60 kDa SSA/Ro antibodies supporting an autoimmune etiology as the most likely cause of the child's AV block, although other contributing factors cannot be entirely excluded.

Notably, the dual electrode strategy with regular switching between wire pairs every few days was effective in maintaining consistently low pacing thresholds. Over the course of 90 days, only a very moderate threshold increase from 2.8 mV to 5.2 mV was observed, allowing safe somatic growth and precluding the need for early permanent pacemaker implantation.

On day 90, weighing 2,890 g, a permanent epicardial pacemaker system was implanted. Through an inferior partial sternotomy, a bipolar ventricular lead (Medtronic CapSure Epi 4968, 25 cm) was affixed to the diaphragmatic and free wall of the right ventricle. The generator (Microny II SR + VVI; Abbott, USA) was placed in a subcutaneous pocket in the upper left abdominal wall (Figure 1B). Piperacillin/Tazobactam was administered for five days postoperatively. Further course was uneventful, with no signs of wound infection or dehiscence.

By day 118, she was discharged home in good clinical condition. At discharge, her weight was 3,455 g (50th percentile), head circumference measured 36 cm (75th percentile), and length was 53.8 cm (75th percentile). The pacemaker was functioning reliably at a set rate of 100 beats per minute, with stable pacing thresholds.

## Discussion

Congenital complete atrioventricular (AV) block in extremely low birth weight (ELBW) infants presents a rare and complex clinical challenge due to physiological immaturity, surgical constraints, and high comorbidity rates. This case describes the most premature and lowest-weight infant to date—born at 25 + 2 weeks' gestation, weighing 740 g—to undergo successful staged epicardial pacemaker implantation.

Prior literature reports only five cases of pacemaker implantation in infants born between 26- and 28-weeks' gestation, with two resulting in neonatal mortality (5–10). Compared to these, our case pushes the boundaries of current neonatal pacing capabilities.

Although systematic data on the longevity of single-site epicardial temporary pacing in ELBW infants are limited, previous studies have reported increasing pacing thresholds and fibrotic encapsulation within 1–2 weeks of implantation, which may compromise pacing efficacy (4). To mitigate this, we employed a dual-site epicardial pacing strategy, alternating the use of electrode pairs every few days to reduce localized fibrosis and preserve myocardial capture. This approach maintained low pacing thresholds over a 90-day period (rising only moderately from 2.8 to 5.2 mV), enabling safe deferment of permanent pacemaker implantation until the infant reached nearly 3 kg. To our knowledge, this dual-site technique has not been widely reported in neonates, and its success in this case suggests that alternating electrode sites may prolong capture efficacy and delay the need for permanent implantation in high-risk preterm infants.

A notable aspect of our approach was the use of dual-site temporary epicardial pacing wires, allowing alternating electrode use to minimize local fibrosis and maintain low capture thresholds over 90 days.

Despite the added complexity of hydrops fetalis, grade III intraventricular hemorrhage, hydrocephalus requiring shunting, and intestinal perforation, the outcome was favorable. This underscores the importance of individualized pacing strategies, early multidisciplinary involvement, and a staged approach in managing high-risk neonates with complete AV block.

In conclusion, this case pushes the boundaries of neonatal cardiac care by demonstrating that dual-site temporary epicardial pacing followed by delayed permanent implantation is feasible even in the most premature infants. It underscores the potential to expand current pacing criteria through individualized, staged approaches in ELBW neonates.

## Data Availability

The datasets presented in this article are not readily available because of ethical and privacy restrictions. Requests to access the datasets should be directed to the corresponding author.
